# Management of Patent Ductus Arteriosus in Premature Infants in 2020

**DOI:** 10.3389/fped.2020.590578

**Published:** 2021-02-11

**Authors:** Sarah Parkerson, Ranjit Philip, Ajay Talati, Shyam Sathanandam

**Affiliations:** ^1^Department of Pediatrics, University of Tennessee, Memphis, TN, United States; ^2^Division of Pediatric Cardiology, University of Tennessee, Memphis, TN, United States; ^3^Division of Neonatology, University of Tennessee, Memphis, TN, United States

**Keywords:** PDA, prematurity and low birth weight, transcatheter ductus closure, patent ductus arteriosus (patent arterial duct), surgical ligation of patent ductus arteriosus

## Abstract

The patent ductus arteriosus (PDA) is the most commonly found cardiac condition in neonates. While there have been several studies and thousands of publications on the topic, the decision to treat the PDA is still strongly debated among cardiologists, surgeons, and neonatologists. This is in part due to the shortage of long-term benefits with the interventions studied. Practice variations still exist within sub-specialties and centers. This article briefly summarizes the history, embryology and histology of the PDA. It also succinctly discusses the hemodynamic significance of a PDA which builds the framework to review all the available literature on PDA closure in premature infants, though not a paradigm shift just yet; it introduces transcatheter PDA closure (TCPC) as a possible armament to the clinician for this age-old problem.

## Introduction

The patent ductus arteriosus (PDA) is the most common cardiac condition affecting neonates (1). While there has been several studies and thousands of publications on the topic, the decision to treat the PDA is still strongly debated among cardiologists, surgeons, and neonatologists (2–4).

Numerous studies have found an association with untreated PDAs and significant neonatal morbidities (5–7). When left untreated, the PDA and resulting left-to-right shunt can compromise systemic perfusion. Determining the volume of the shunt is a crucial step in deciding the course of action for premature infants with a PDA. Physicians use their clinical assessment, echocardiography, and indicators of systemic hypoperfusion or pulmonary over-circulation in order to quantify the shunt but this process has not been standardized (8, 9) and thus varies across institutions. While increased morbidity is associated with PDA, the management options have been linked to adverse outcomes (10–12); which leads to debate over whether or not to treat the PDA (2). Furthermore, if and when a PDA needs to be treated, how do we treat it? (13) This chapter intends to outline the current literature about the embryology, pathophysiology, and treatment approaches for the PDA in the premature infant in 2020.

## History

The PDA was the first congenital heart lesion that was surgically repaired. This was performed by Dr. Robert Gross in 1938 (14). In the Second century, Galen was one of the first to describe a notion of blood traveling between the heart and the lungs. This concept was later developed into a complete anatomical and physiologic description of PDA many centuries later. In 1989, Krichenko et al. (15) angiographically classified the PDA based on the ductal lumen at the aortic and pulmonary ends. Closure of the PDA in a premature neonate with respiratory distress syndrome (RDS) was first detailed by Dr. ML Powell in 1963 (16). With the increasing survival of preterm neonates, the consequent increase in PDA closures in preterm infants and the special morphologic characteristics of the preterm ductus, the PDA was reclassified in 2016 to aid with the choice of device used for transcatheter device closure in this population (17).

## Embryology

At the early embryonic stage, the ductus arteriosus (DA) is present on the right and left sides but the right atrophies between 37 and 40 days of embryonic gestation (18). The DA is created from the same embryonic structure that makes the pulmonary artery: the left 6th aortic arch. The DA attaches to the inner curve of the arch, distal to the left subclavian artery. There is a high resistance within the pulmonary vasculature (19) during development of the fetal lungs. Due to this degree of resistance, blood travels from the left pulmonary artery, through the DA, and into the descending aorta which preserves right ventricular function (20). If the DA were to close prematurely *in utero*, the right ventricular afterload increases and the fetus is at risk to develop right heart failure and fetal hydrops (21).

## Histology

Histologically, the walls of the DA are mainly muscular in contrast to the walls of the adjacent aorta and pulmonary artery, which are fibro-elastic (22). The DA is comprised of smooth muscle fibers which are arranged in longitudinal and spiral layers and surrounded by concentric layers of elastic tissue. The great arteries are composed primarily of elastic fibers arranged circumferentially. After birth, the medial smooth muscle fibers contract in response to the exposure to oxygen-rich ambient air (23). This leads to constriction of the lumen and shortening of the DA length which begins at the pulmonary end until there is functional closure between 24 and 48 h. The second stage of closure involves proliferation of the medial and intimal connective tissue and smooth muscle atrophy which leads to the conversion of a muscular vessel into a ligamentous non-contractile structure, the ligamentum arteriosum over the next 3 weeks (24). Vascular endothelial growth factor (VEGF) has been thought to be involved in DA closure. However, VEGF polymorphism rs2010963 status has not been shown to affect PDA incidence or successful treatment with cyclooxygenase inhibitors in preterm infants (25).

## Physiology

The predominant cardiac output in fetal life bypasses the lungs via right to left shunting at the DA. This is possible due to the low systemic vascular resistance from the placenta and the high pulmonary vascular resistance in the lungs. Systemic vascular resistance is increased after birth when the cord is clamped (26). In addition, due to ventilation, the pulmonary vascular resistance is decreased and pulmonary blood flow is increased. This causes blood to shunt left to right through the DA. In term infants, predominant left to right shunting occurs within 10 min and is entirely left to right within 24 h of life (27).

## PDA in the Premature Neonate

The incidence of PDA is inversely related to the gestational age. It remains open at 4 days of age in 10% of neonates born at 30–37 weeks, 80% of those between 25 and 28 weeks gestation and 90% born <24 weeks gestation (28–31). In these extremely premature infants, the PDA can persist for weeks, in comparison to infants born after 28 weeks in which the PDA is more likely to spontaneously close (32). Failure of closure of the DA in these infants is more commonly due to issues associated with prematurity rather than aberrations of the DA itself (33). The pressure gradient between the aorta and pulmonary artery (PA) as well as the size and resistance of the DA determine the hemodynamic effects of the PDA. The vessel's length, internal diameter, and intrinsic elastance properties define the resistance within the DA (34). Whereas, a small PDA may be left untreated without complications, a large PDA left open long-term can lead to pulmonary hypertension (19, 35). The role of platelet function in the spontaneous PDA closure in the preterm infant has been studied (36, 37). Lower counts of mature platelets are an independent predictor of a hsPDA (38).

The shunting of blood from left-to-right via the PDA causes pulmonary over-circulation and left-sided volume overload in the heart. This increase in pulmonary blood flow can affect the maturation of the pulmonary vasculature in a premature infant (33). It can result in pulmonary edema, reduced lung compliance, and ineffective gas exchange. This, in turn, leads to increased ventilator settings, which can cause parenchymal lung damage in the neonate (39). It has been suggested that PDA closure within the first 4 weeks of life may allow for faster weaning of oxygen and ventilatory support in extremely low birth weight (ELBW) infants (birth weight ≤ 1,000 g) (40); however, further investigation is needed.

With severe volume overload in the left heart, the end-diastolic pressure of the left atrium and ventricle can be elevated. The left ventricular myocardium undergoes remodeling to a larger and more spherical shape (41). The larger the left-to-right shunt, the more association with risk of necrotizing enterocolitis, intraventricular hemorrhage, acute kidney injury and death (7, 42, 43). Very low birth weight preterm infant survivors of pulmonary hemorrhage were associated with a PDA (79%) in comparison to their controls (55%) (44). Although, this mechanism is not completely understood. One widely accepted theory is that the shunting through the PDA results in lower systemic diastolic pressure and thus threatens end-organ perfusion. Others blame the morbidities of PDA on reperfusion injury suggesting that the left ventricle becomes more compliant overtime and thus increases its cardiac output. The reperfusion that occurs after a phase of hypoperfusion is thought to be the culprit of the end-organ damage in the premature neonate (45).

There is still widespread discordance about best management of a PDA and the timing in which to apply said management (4, 46, 47). A survey in 2018 found many disparities between neonatologists and cardiologists concerning the management of PDA in neonates (2). Most cardiologists believe that PDA closure alters the clinical outcomes in infants born <28 weeks' gestation, while almost half neonatologists surveyed disagree with this statement (2). There is currently not enough evidence to determine whether systemic complications associated with hemodynamically significant PDA in ELBW infants is due to the PDA or simply the result of prematurity (48).

## Defining A Hemodynamically Significant PDA

There is a larger body of epidemiological data that reveals an association between a PDA and multiple morbidities including intraventricular hemorrhage (IVH), necrotizing enterocolitis (NEC), retinopathy of prematurity (ROP), and bronchopulmonary dysplasia (BPD) (5, 49, 50). However, significant differences have not been demonstrated in randomized trials of PDA closure. Prior to the twenty-first century, almost all PDAs were arbitrarily deemed hemodynamically significant and physicians aimed to close them soon after birth (51). That approach is no longer favored, and the majority of physicians prefer to watch and wait (46). The lack of quantifiable improvement in outcomes after PDA closure likely influenced this practice change. The outcomes without noticeable improvement after PDA closure include BPD, NEC, IVH, ROP, periventricular leukomalacia, long-term neurodevelopmental status, and death (52, 53).

**Figure 1:**
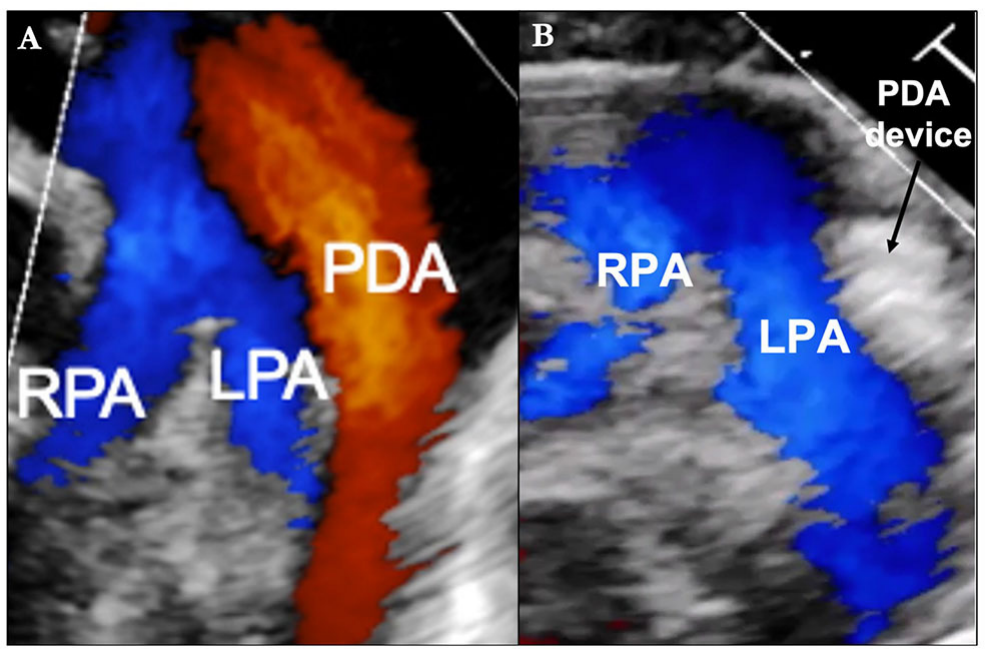
**(A)** Transthoracic Echocardiogram (parasternal short axis image with color Doppler) demonstrating the relative size of the patent ductus arteriosus (PDA) in comparison to the left pulmonary artery (LPA) and right pulmonary artery (RPA). **(B)** Post-transcatheter PDA closure.

**Figure 2:**
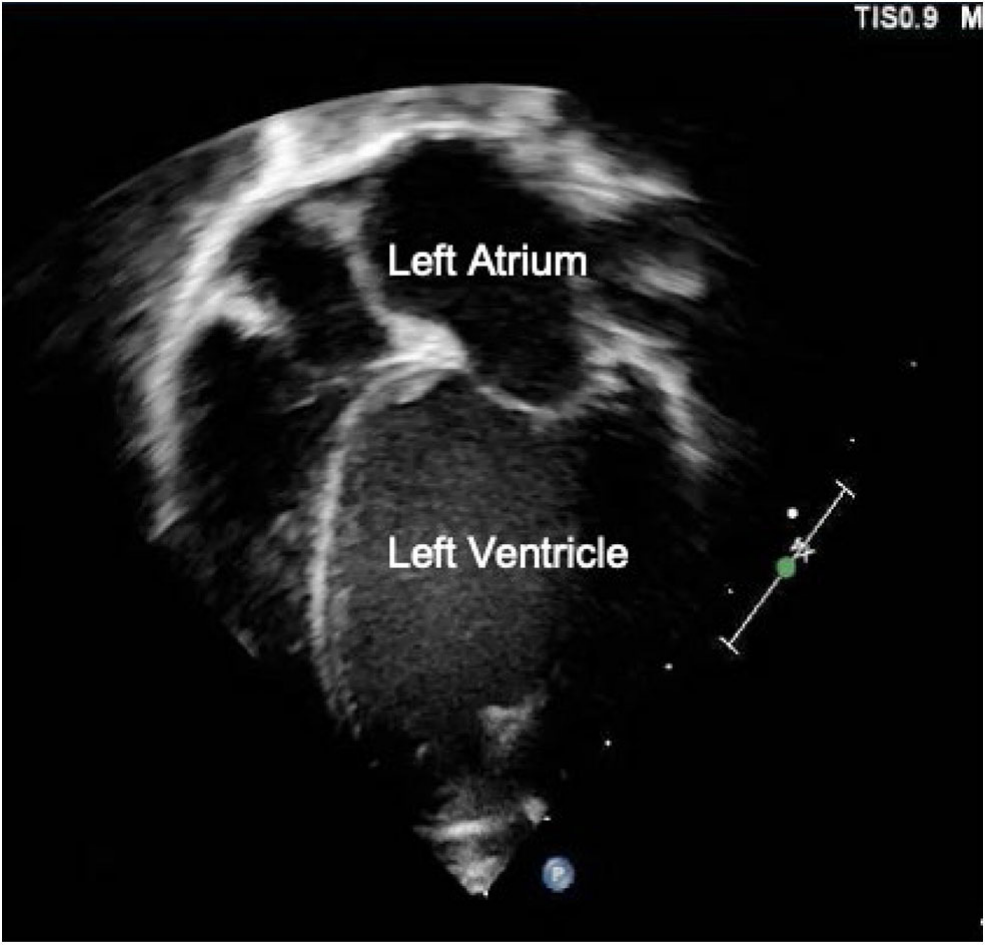
Transthoracic Apical 4-chamber view showing left atrial and ventricular dilation suggestive of a hemodynamically significant PDA.

**Figure 3:**
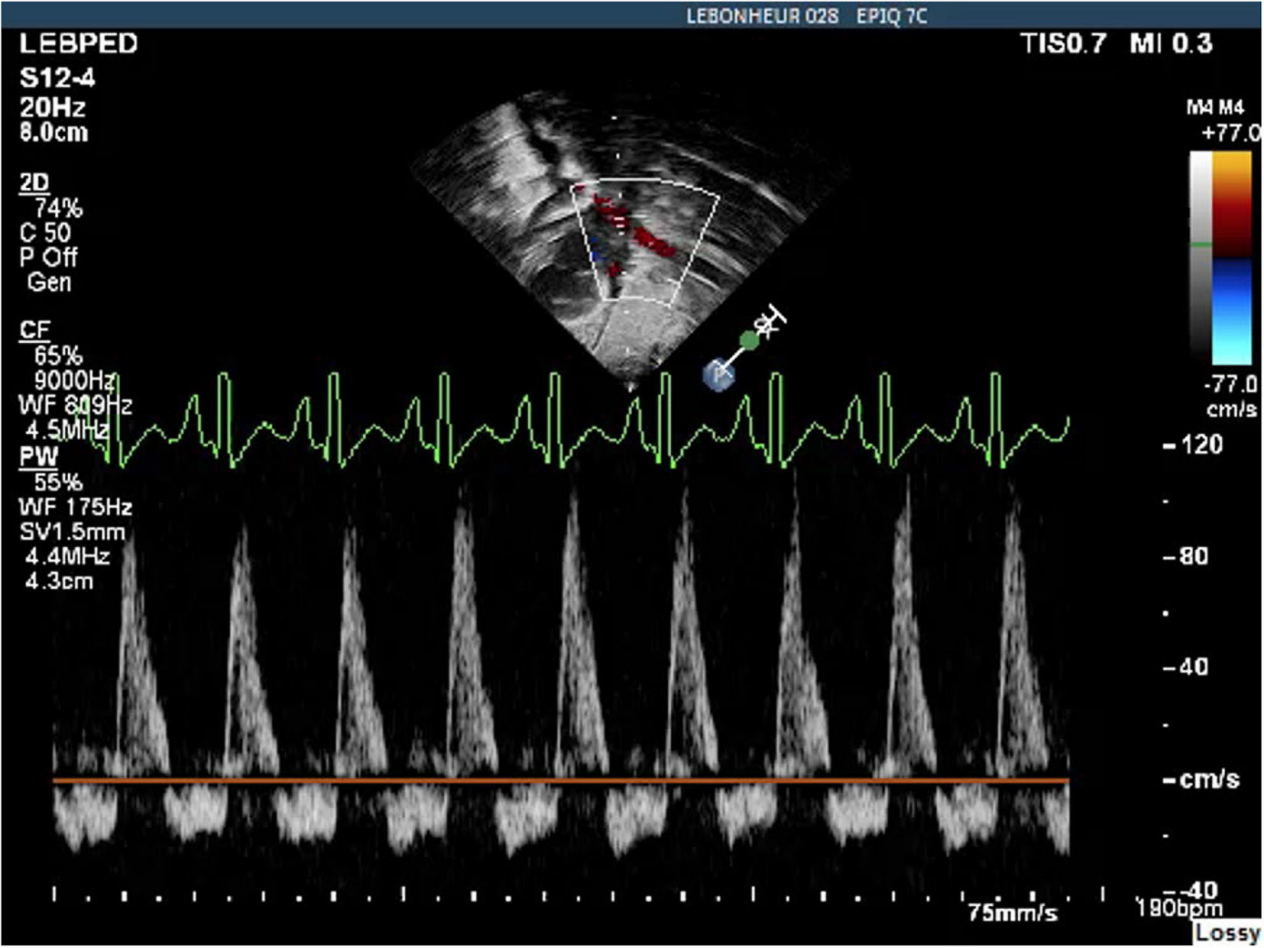
Abdominal Aorta Doppler pattern showing diastolic flow reversal indicative of a hemodynamically significant PDA.

Echocardiography has been examined in multiple studies as an objective measure of a hemodynamically significant PDA (hsPDA) (49, 54, 55). The echocardiographic parameters used in determining if a PDA is hemodynamically significant include the transductal diameter, evidence of left-sided volume overload, quantification of ductal shunting, and magnitude of pulmonary overcirculation (56). The transductal diameter and the flow pattern (low velocity flow) are used to quantify the shunt size as seen in Figure 1A (57). Indices of left-sided volume overload and pulmonary over-circulation are closely related and thus use many of the same indices for evaluation as seen in Figure 2. Albeit dated, one of the most used measurements to determine is a PDA is hemodynamically significant is the ratio of the left atrium diameter (LA) to the aortic root diameter (Ao) by M-mode echocardiography. If the LA:Ao ratio is >1.4, there is likely left-sided volume overload secondary to increased blood return from the lungs. Another important echocardiographic measurement includes reversal of flow in diastole in the abdominal aorta suggestive of a substantial shunt [i.e., evidence of systemic steal (Figure 3)]. Individually, these indices are not specific to a PDA but when presenting together in a premature infant with RDS, it is reasonable to believe there is a hemodynamically significant PDA present. Although there are numerous suggested protocols for assessment of a hsPDA (49, 50, 58–61), there is no validated protocol that confirms the echocardiographic information with real-time hemodynamic information from cardiac catheterization.

Echocardiography can also be useful as a predictive measure of whether a PDA will become hemodynamically significant. One study found that the ductal anatomy during the first 12 h of life, specifically a ductal length <5.2 mm, was predictive of a hsPDA by 72 h of life with a sensitivity of 83% and a specificity of 86% (62).

Multiple publications have investigated serum biochemical markers as indicators of a hsPDA both independently and with echocardiography. These markers include B-type natriuretic peptide (BNP), amino-terminal pro-B-type natriuretic peptide (NT-proBNP), and cardiac Troponin T (cTnT) (63, 64). There are also urinary markers such as Neutrophil gelatinase-associated lipocalin and heart-type fatty acid binding protein that have been used to predict ductal closure timing (65). While locally certified BNP or NT-proBNP assays can be useful to trend for appraisal of response to therapy, the variability of assays across institutions prevents universal assertions about their utilization (56). A study of premature neonates did find a correlation between the cTnT levels and the ductal diameter, LA:Ao ratio and descending aortic end-diastolic velocity (57). In addition, these biomarkers may help clinicians determine the effect of their treatments on infants' cardiac function. For example, one study found that even 2 weeks after treatment with oral ibuprofen, urinary NT-proBNP was still elevated, suggesting that there is still an effect on cardiac function even when the infant is clinically stable post-treatment (66).

Clinical judgment can be another useful resource in the decision to treat a PDA. One study found that presence or absence of certain clinical symptoms was appropriate for determining treatment of PDA and may prevent unnecessary medical treatments in this vulnerable population (67). Alone, echocardiographic indices and serum biochemical markers cannot reliably determine if a PDA is hemodynamically significant. However, when used in combination with clinical data, practitioners can make that determination on a case-by-case basis (9).

## Prophylactic Closure of PDA

Though the presence of a moderate to large PDA has been associated with morbidity, as demonstrated in over 60 controlled trials, the lack of any decrease in morbidity and mortality with ductal closure has questioned the utility of prophylactic PDA closure in premature infants (68, 69). There are four possible explanations as described by McNamara for the lack of proven clinical benefit after ductal closure (70). First, the “hemodynamically significant” PDA has no true causal relationship with adverse outcomes. Second, the conclusions of many studies are unreliable due to the variable definition of “hemodynamically significant” and therefore results have been diluted by “hemodynamically non-significant” PDAs. Third, frequent cross-over between placebo and treatment groups may have obscured any visible benefits. Fourth, there is a causal relationship between the hsPDA and adverse outcomes but closure alone will not fully ameliorate the morbidities or mortalities. The multiple confounding factors that coexist may explain why the trials of prophylactic closure have shown only some reduction in various morbidities (52, 71).

Prophylactic medical closure of the PDA before the PDA becomes “hemodynamically significant” has been shown to reduce the incidence of severe IVH and the need for surgical ligation however it has not been shown to reduce BPD, NEC, or improve long-term neurodevelopmental outcomes (72). It is unclear why the decreased incidence of severe IVH has not translated into enhanced neurodevelopmental outcomes in the long-term. The Ment-trial in 1994 showed favorable neurodevelopment outcomes in 4.5 and 8-years old children who were treated with Indomethacin prophylaxis (73). However, the PDA-TOLERATE trial found medical treatment after the 1st week of life was not associated with reduced PDA ligations or PDAs present at discharge in infants born before 28 weeks gestation (74). In addition, the infants born between 26 and 28 weeks gestation had increased rates of late-onset sepsis and death when exposed to early medical treatment suggesting that early medical treatment is not without long-term negative effects (74). This has led many neonatologists to adopt a conservative approach, which compared to medical treatment, has resulted in similar rates of NEC, IVH, and ROP (75). In one study, premature infants who did not get treatment for their PDA had lower rates of mortality than the infants who received medical treatment (75). Spontaneous closure of PDAs and the need for rescue treatment for those in the conservative treatment group make it difficult to fully evaluate the morbidities associated with conservative treatment (76).

Prophylactic surgical ligation of PDA was a common practice before medical therapy was widely available. In 1989, Cassady published the first randomized controlled trial of prophylactic surgical ligation and found a significant reduction in rate of NEC but no reduction in mortality, severe IVH, BPD, or ROP (77). Due to the significant morbidities associated with surgical ligation, prophylactic surgical ligation is no longer an accepted practice (78, 79).

## Medical Closure of A Hemodynamically Significant PDA

Both indomethacin and ibuprofen are accepted by the US Food and Drug Administration (FDA) for closing a PDA (80, 81). These drugs prevent the transition of arachidonic acid to prostaglandin by inhibiting the cyclooxygenase site of the prostaglandin synthase enzyme, thus causing constriction of the PDA. Ibuprofen can be as successful as indomethacin for closure of the PDA. In addition, ibuprofen offers a lower risk of NEC and acute kidney injury (82). The long-term benefits of using ibuprofen compared to indomethacin has not been extensively studied and thus the data is not currently available.

Ibuprofen is given in three doses 24 h apart. The standard dosing is 10 mg/kg for the first dose and half (5 mg/kg) for the second and third doses (83). However, new research has found a higher closure rate when the ibuprofen dose is doubled (54.8% closure compared to 30.6%) (84, 85). Mitra published a meta-analysis in 2018 which also observed a higher probability of PDA closure and no increase in adverse outcomes when the dose was doubled (86). Another RCT using early oral ibuprofen prophylaxis showed reduced rates of hsPDA albeit not statistically significant and with a high prevalence of adverse events (intestinal perforation and kidney injury) (87). This is further support by a study that found lower serum ibuprofen levels in premature infants whose hsPDA did not close after 3-dose treatment; specifically, the trough after the first dose was significantly lower compared to the group of infants whose hsPDA did close after 3-dose treatment (88).

The preferred route of ibuprofen administration has just been clarified by recent studies. A Cochrane review in 2015 observed that ibuprofen administered intravenously was less effective at PDA closure than ibuprofen administered orally (RR 0.37; 95% CI 0.23–0.61) (65). This was also seen in a large network meta-analysis (74). Pacifici suggested that the slower absorption and prolonged half-life with oral administration offered more time for the drug to interact with the PDA and thus made it more effective (89). However, a study of ELBW infants found that those infants who received oral ibuprofen for PDA closure had a higher rate of PDA reopening compared to those who received IV ibuprofen (90). In terms of safety, evidence supports that oral ibuprofen is as safe as IV ibuprofen in very low birth weight infants (91). Furthermore, one study observed that neurodevelopmental outcomes in premature infants who received oral ibuprofen vs. IV ibuprofen for PDA closure were not significantly different (92). Ibuprofen has been associated with higher risk of NEC, spontaneous intestinal perforation, and acute kidney injury (81) In addition, decreased blood flow to the kidneys, mesentery, and brain was revealed through near infrared spectroscopy (NIRS) and Doppler ultrasonography after indomethacin/ibuprofen was given for PDA closure (93–97). Consequently, withholding enteral feedings has become more popular when administering indomethacin/ibuprofen to premature infants in order to reduce the adverse gastrointestinal outcomes. However, some studies have investigated this and did not find a disparity in enteral feeding tolerance or harmful events involving the gastrointestinal tract when infants were given smaller volume enteral feeds during indomethacin/ibuprofen therapy (98, 99).

Recently, acetaminophen has been suggested as a substitute for indomethacin and ibuprofen. The US FDA has not approved the use of acetaminophen in neonates to close the PDA. The mechanism of acetaminophen is not completely known, but it is thought to inhibit the peroxidase section of the prostaglandin synthase enzyme causing narrowing of the PDA (100). Oral acetaminophen has not been extensively studied like the cyclooxygenase inhibitors, but was thought to be as successful as oral ibuprofen in closing a PDA (101). In addition, the incidence of renal insufficiency was reduced with oral acetaminophen administration compared to high dose oral ibuprofen (102). As seen with ibuprofen, one study found a lower serum acetaminophen level in premature infants whose PDA did not close after a 3-days course of acetaminophen (103). With potential for effective closure of the PDA and reduced adverse events (104), acetaminophen has become an appealing alternative to cyclooxygenase inhibitors (105), especially in infants with other morbidities easily aggravated by indomethacin/ibuprofen (106). However, a recent multicenter randomized control trial evaluating the effectiveness of IV acetaminophen over IV indomethacin in infants <32 weeks gestation with a birthweight ≤ 1,500 gms with a hemodynamically significant PDA by echocardiogram showed reduced effectiveness (5.9 vs. 55% closure rate) (107). In addition, a RCT evaluating the impacts of paracetamol compared to ibuprofen on neurodevelopment outcomes at 18–24 months corrected gestational age showed no significant difference (108). A single center trial of 12 infants evaluating the role of combined (paracetamol and ibuprofen) therapy to monotherapy-resistant hsPDA showed a 75% success rate (109). However, in a single center double-blinded randomized controlled pilot study, there was no benefit observed with adding IV paracetamol to IV ibuprofen for the treatment of hsPDA (110).

## Surgical Closure of A Hemodynamically Significant PDA

The first RCT evaluating surgical ligation vs. conservative “standard” medical care was done by Cotton and colleagues who tested ventilated VLBW neonates at 8–10 days of life. Albeit small, the study showed that routine early surgical ligations led to earlier extubations and a lower hospital bill (111). Unfortunately, surgical ligation has frequently been linked to increased morbidities including BPD, ROP, and long-term neurodevelopmental impairment (10, 112–116). The TIPP trial (trial of indomethacin prophylaxis in preterm infants) also corroborated that patients who underwent surgical ligation had more BPD, severe ROP, and 18–22 months neurosensory deficiency in comparison to patients who had medical therapy alone (72).

However, there are many confounding variables linked to surgical ligation that complicate the reasons for neurodevelopmental deficiency. It is unclear whether the outcomes are purely cause-and-effect or if surgery is an indication of clinical severity. With substantial adverse events depicted after surgical ligation (117–123) and the debate over whether a PDA even needs to be closed, most neonatologists err on the side of caution and defer surgical ligation even if medical management fails (2, 46).

## Percutaneous Closure of Hemodynamically Significant PDA

Transcatheter PDA closure (TCPC) has come to be the treatment of choice in adults and children (20). However, there is still hesitation with patient selection and timing of procedure due to opposing results on the safety of TCPC during infancy (124). The largest meta-analysis to date assessing infants undergoing percutaneous closure of their PDA was published by Backes in 2017. In 635 procedures within 38 studies, 92.2% were successful closures and the rate of adverse events was 23.3%, with only 10.1% being “clinically significant (125).” In 2016, Zahn evaluated TCPC in the extremely premature population, which included 24 infants with a mean weight of 1,249 grams. His successful closure rate was 88% and his adverse event rate was 12.5%. At follow up (median time of 11 months), all 24 patients were alive and did not have residual PDA, LPA stenosis, or aortic coarctation (126). At our institution, TCPC in premature infants had a significantly lower complication rate compared to surgical ligation (3.3 vs. 25.7%) (127, 128). We have currently performed TCPC on 108 infants weighing <1,000 grams at our institution. The complications in the 241 infants weighing between 500 and 2,000 grams at the time of the TCPC at our institute included: coarctation of the aorta (2), pericardial effusion needing drainage (2), tricuspid valve injury (1), one procedure related mortality early in the experience, device embolization and retrieval (1) and left pulmonary artery stenosis requiring device retrieval (1). In contrast, the surgical group (*n* = 167) had two procedure related mortalities, wound dehiscence (2), residual PDA requiring re-intervention (4), Pneumothorax (3), Sepsis/pneumonia (4), left ventricular wall motion abnormality needing digoxin at discharge (1), worsening respiratory status (2), and post-ligation syndrome (25).

While percutaneous PDA closure can be done at bedside using echocardiography and portable fluoroscopy, transporting the premature infant to the catheterization lab may be necessary. This transport requires thorough coordination since ELBW infants often require temperature and ventilatory regulation (129). In addition, there are some procedural alterations that can be made to better accommodate the premature infant during percutaneous PDA closure.

Since limb ischemia has been associated with accessing small diameter arteries in ELBW infants, the procedure is performed from a venous access only (130, 131). Femoral vessel nomograms allow for pre-procedure planning in certain settings but they may not include premature infants in the data set so it may be safest to avoid arterial access in premature infants (132). Reducing overall procedure time is especially important in premature infants and can be done by minimizing intracardiac catheter manipulation, contrast dosing, and unnecessary hemodynamic measurements (133, 134). Equally important is ensuring optimal device placement prior to device deployment (135). Without having arterial access, aortography is not possible so a transthoracic echocardiogram (TTE) should be used for evaluation of device placement as seen in Figure 1B (135). If a different device size is required due to inadequate placement or residual shunting, the initial device can be retrieved via catheter and a new device placed all within the same procedure setting (136). The risks associated with percutaneous PDA closure, while low even in ELBW infants, include device embolization, cardiac perforation, aortic coarctation, and LPA obstruction (125).

Currently, there are a number of PDA closure devices available for infants and children >5 kg. However, the DA morphology differs in ELBW infants in that it is long and tortuous (17) compared to older infants and children, which can compromise the effectiveness of these devices in ELBW infants (137). Recently, a new device was FDA approved for PDA closure in infants ≥ 700 g (138). The success rate with this new device was 95.5% (191/200) in all patients and 99% (99/100) in patients ≤ 2 kilograms. Of implanted patients, 99.4% reached the primary effectiveness endpoint. A primary safety endpoint event was reached in four patients (two transfusions, one hemolysis, and one aortic obstruction). No branch pulmonary artery obstructions were observed. Worsening of tricuspid regurgitation (TR) was found in five patients after the procedure. This study clearly showed safety and efficacy of TCPC even though it was restricted to 10 centers. It adds to the armament of the neonatologist in the early treatment of PDAs in premature infants and may encourage further investigation on the outcomes of early PDA closure in this population (138).

With growing experience, we have found that early PDA closure may afford better overall clinical outcomes in ELBW infants. In the past, early surgical ligation of the PDA did not demonstrate any benefit. However, unlike surgical ligation, TCPC involves no cutting or stitching to the infants' chest, there is no handling of the premature lungs and no inflammatory surge. Therefore, TCPC may be overall beneficial. It is similar to surgical ligation in that it affords immediate, definitive closure of the PDA unlike medical therapy, which is not 100% effective and requires a long time period to work. While TCPC is a technically feasible and safe therapeutic option, the lack of comparative trials and widespread operator experience, prevents it from being the current standard of care. Since ELBW infants who have undergone TCPC usually have multiple co-morbidities and are at high risk for hospital readmission during their 1st year of life, it is important to have a multidisciplinary follow-up visit after initial discharge to monitor their progress (139, 140). However, given the concerns of surgical ligation during infancy (52–55, 117–123), TCPC represents a potentially attractive alternative especially when medical therapy has failed.

## Post-Ligation Syndrome

Substantial hemodynamic changes take place after ligation of PDA and they have only recently started being explored in a scientific manner. A sudden increase in afterload and a decrease in left ventricular (LV) preload occurs following ligation, putting the neonate at high risk for decreased cardiac output. In fact, decreased LV output has been reported in up to 50% of premature neonates after PDA ligation (141). This cardiac deterioration is termed post-ligation syndrome and can occur relatively quickly. Signs of a low cardiac output state include hypotension, increased metabolic acidosis and respiratory failure (142, 143). Studies have found a significantly lower incidence of post-ligation syndrome in ELBW infants who underwent transcatheter PDA closure compared to surgical ligation (144–146). Jain demonstrated that prophylactic milrinone administration reduced the incidence of post-ligation syndrome from 44 to 11% in high-risk patients (147). In addition, calcium chloride administration following transcatheter PDA closure can also reduce the incidence of post-ligation syndrome in ELBW infants (148).

## Conclusion

Management of the PDA continues to be an elusive challenge for neonatologists and pediatric cardiologists despite it being the most common cardiac condition affecting premature neonates. After 50 years of scientific investigation yielding thousands of publications, there is still no agreement on the definition of a hemodynamically significant PDA and how best to treat it. However, many clinical lessons have been brought to light and cannot be ignored. We know that routine PDA closure soon after birth does not diminish the morbidities associated with a PDA. In addition, surgical ligation has been associated repeatedly with higher occurrence of BDP, ROP, and poorer neurodevelopmental outcomes. When medically treating the PDA, we have realized several medications which have had positive results, some with more adverse side effects than others. With the introduction of percutaneous PDA closure, we have the potential to be less invasive; however, as in most treatment modalities, the risks remain. Pharmacogenetics appears to be the next frontier as we contemplate the continued questions of who needs intervention for their hsPDA and how best to achieve this while minimizing the morbidity and mortality, specifically in premature infant population. With continued exploration, we continue to adapt our theories about the persistent ductus arteriosus and its effects.

## Author Contributions

All authors were involved in substantial contributions to the conception, design of the manuscript, drafting the article, and revising it critically for important intellectual content.

## Conflict of Interest

The authors declare that the research was conducted in the absence of any commercial or financial relationships that could be construed as a potential conflict of interest.

## References

[1] Mitchell SC Korones SB Berendes HW. Congenital heart disease in 56,109 births: incidence and natural history. Circulation. (1971) 43:323–32. 10.1161/01.CIR.43.3.323 5102136

[2] Sathanandam S Whiting S Cunningham J Zurakowski D Apalodimas L Waller BR. Practice variation in the management of patent ductus arteriosus in extremely low birth weight infants in the United States: survey results among cardiologists and neonatologists. Congenit Heart Dis. (2019) 14:6–14. 10.1111/chd.12729 30811803

[3] Bose CL Laughon M Treatment to prevent patency of the ductus arteriosus: beneficial or harmful? J Pediatr. (2006) 148:713–4. 10.1016/j.jpeds.2006.03.015 16769371

[4] Benitz WE. Committee of the fetus and newborn. Patent ductus arteriosus in preterm infants. Pediatrics. (2016) 137:e20153730. 10.1542/peds.2015-3730 26672023

[5] Noori S McCoy M Friedlich P Bright B Gottipati V Seri I. Failure of ductus arteriosus closure is associated with increased mortality in preterm infants. Pediatrics. (2009) 123:e138– 44. 10.1542/peds.2008-2418 19117835

[6] Hamrick SE Hansmann G. Patent ductus arteriosus of the pre- term infant. Pediatrics. (2010) 125:1020–30. 10.1542/peds.2009-3506 20421261

[7] Dollberg S Lusky A Reichman B. Patent ductus arteriosus, indomethacin and necrotizing enterocolitis in very low birth weight infants: a population-based study. J Pediatr Gastroenterol Nutr. (2005) 40:184–8. 10.1097/00005176-200502000-00019 15699694

[8] El-Khuffash A Levy PT Gorenflo M Frantz ID. The definition of a hemodynamically significant ductus arteriosus. Pediatr Res. (2019) 85:740–1. 10.1038/s41390-019-0342-7 30770863

[9] Bischoff AR Giesinger RE Bell EF McNamara PJ. Precision medicine in neonatal hemodynamics: need for prioritization of mechanism of illness and defining population of interest. J Perinatol. (2020) 40:1446–9. 10.1038/s41372-020-0741-y 32719495

[10] Clyman R Cassady G Kirklin JK Collins M Philips JB III. The role of patent ductus arteriosus ligation in bronchopulmonary dysplasia: reexamining a randomized controlled trial. J Pediatr. (2009) 154:873–6. 10.1016/j.jpeds.2009.01.005 19324366 PMC2709418

[11] Sung SI Chang YS Chun JY Yoon SA Yoo HS Ahn SY. Mandatory closure versus nonintervention for patent ductus arteriosus in very preterm infants. J Pediatr. (2016) 177:66–71. 10.1016/j.jpeds.2016.06.046 27453374

[12] El-Mashad AE El-Mahdy H El Amrousy D Elgendy M. Comparative study of the efficacy and safety of paracetamol, ibuprofen, and indomethacin in closure of patent ductus arteriosus in preterm neonates. Eur J Pediatr. (2017) 176:233–40. 10.1007/s00431-016-2830-7 28004188

[13] Whiting S Sathanandam S Patent ductus arteriosus in extremely low birth weight infants - if, when and how to close it? In: Barría RM, editor. Neonatal Intensive Care Unit. Croatia: IntechOpen (2020). Available online at: https://www.intechopen.com/books/update-on-critical-issues-on-infant-and-neonatal-care/pda-closure-in-elbw-infants-if-when-and-how-to-do-it

[14] Gross RE Hubbard JP Surgical ligation of a patent ductus arteriosus: a report of first successful case. JAMA. (1939) 112:729–31. 10.1001/jama.1939.02800080049011 6363741

[15] Krichenko A Benson LN Burrows P Moes CA McLaughlin P Freedom RM Angiographic classification of the isolated, persistently patent ductus arteriosus and implications for percutaneous catheter occlusion. Am J Cardiol. (1989) 63:877–80. 10.1016/0002-9149(89)90064-7 2929450

[16] Merritt TA DiSessa TG Feldman BH Kirtkpatrick SE Gluck L Friedman WF Closure of the patent ductus arteriosus with ligation and indomethacin: a consecutive experience. J Pediatr. (1978) 93:639–46. 10.1016/S0022-3476(78)80909-3 279657

[17] Philip R Waller BR 3rd Agrawal V Wright D Arevalo A Zurakowski D Morphologic characterization of the patent ductus arteriosus in the premature infant and the choice of transcatheter occlusion device. Catheter Cardiovasc Interv. (2016) 87:310–17. 10.1002/ccd.26287 26525611

[18] Congdon ED Transformation of the aortic arch system during the development of the human embryo. Contributions to Embryology. (1922) 14:47–110.

[19] Philip R Johnson JN Naik R Kimura D Boston U Chilakala S. Effect of patent ductus arteriosus on pulmonary vascular disease. Congenit Heart Dis. (2019) 14:37–41. 10.1111/chd.12702 30811787

[20] Schneider DJ Moore JW. Patent ductus arteriosus. Circulation. (2006) 114:1873–82. 10.1161/CIRCULATIONAHA.105.592063 17060397

[21] Leal SD Cavalle-Garrido T Ryan G Farine D Helibut M Smallhorn JF. Isolated ductal closure in utero diagnosed by fetal echocardiography. Am J Perinatol. (1997) 14:205–10. 10.1055/s-2007-994128 9259929

[22] Gittenberger-de Groot AC Moulaert AJ Harinck E Becker AE. Histopathology of the ductus arteriosus after prostaglandin E1 administration in ductus dependent cardiac anomalies. Br Heart J. (1978) 40:215–20. 10.1136/hrt.40.3.215 637973 PMC481984

[23] Reese J. Towards a greater understanding of the ductus arteriosus. Semin Perinatol. (2018) 42:199–202. 10.1053/j.semperi.2018.05.001 29891126 PMC6512790

[24] Fay FS Kooke PH. Guinea pig ductus arteriosus, II: irreversible closure after birth. Am J Physiol. (1972) 222:841– 9. 10.1152/ajplegacy.1972.222.4.841 5027091

[25] Sallmon H Aydin T Hort S Kubinski A Bode C Klippstein T Vascular endothelial growth factor polymorphism rs2010963 status does not affect patent ductus arteriosus incidence or cyclooxygenase inhibitor treatment success in preterm infants. Cardiol Young (2019) 29:893–7. 10.1017/S1047951119001033 31218973

[26] Ovali F. Molecular and mechanical mechanisms regulating ductus arteriosus closure in preterm infants. Front Pediatr. (2020) 8:516. 10.3389/fped.2020.00516 32984222 PMC7477801

[27] van Vonderen JJ te Pas AB Kolster-Bijdevaate C van Lith JM Blom NA Hooper SB. Non-invasive measurements of ductus arteriosus flow directly after birth. Arch Dis Child Fetal Nenatal Ed. (2014) 99:F408–12. 10.1136/archdischild-2014-306033 24966129

[28] Clyman RI Couto J Murphy GM Patent ductus arteriosus: are current neonatal treatment options better or worse than no treatment at all?. Semin Perinatol. (2012) 36:123–9. 10.1053/j.semperi.2011.09.022 22414883 PMC3305915

[29] Weinberg JG Evans FJ Burns KM Pearson GD Kaltman JR. Surgical ligation of patent ductus arteriosus in premature infants: trends and practice variation. Cardiol Young. (2016). 26:1107–14. 10.1017/S1047951115001869 26395077

[30] Heuchan AM Clyman RI. Managing the patent ductus arteriosus: current treatment options. Arch Dis Child Fetal Neonatal Ed. (2014) 99:F431–6. 10.1136/archdischild-2014-306176 24903455

[31] Rolland A Shankar-Aguilera S Diomande D Zupan-Simunek V Boileau P. Natural evolution of patent ductus arteriosus in the extremely preterm infant. Arch Dis Child Fetal Neonatal Ed. (2015) 100:F55–8. 10.1136/archdischild-2014-306339 25169243

[32] Weber SC Weiss K Bührer C Hansmann G Koehne P Sallmon H. Natural history of patent ductus arteriosus in very low birth weight infants after discharge. J Pediatr. (2015) 167:1149–51. 10.1016/j.jpeds.2015.06.032 26239928

[33] Kitterman JA Edmunds LH Jr Gregory GA Heyman MA Tooley WH Rudolph AM. Patent ductus arteriosus in premature infants: incidence, relation to pulmonary disease and management. N Engl J Med. (1972) 287:473–7. 10.1056/NEJM197209072871001 5048708

[34] Tomita H Fuse S Hatakeyama K Chiba S. Epinephrine-induced constriction of the persistent ductus arteriosus and its relation to distensibility. Jpn Circ J. (1998) 62:913–4. 10.1253/jcj.62.913 9890205

[35] Philip R Towbin JA Sathanandam S Goldberg J Yohannan T Swaminathan N Effect of patent ductus arteriosus on the heart in preterm infants. Congenit Heart Dis. (2019) 14:33–6. 10.1111/chd.12701 30811789

[36] Kahvecioglu D Erdeve O Akduman H Ucar T Alan S akir U. Influence of platelet count, platelet mass index, and platelet function on the spontaneous closure of ductus arteriosus in the prematurity. Pediatr Neonatol. (2018) 59:53–7. 10.1016/j.pedneo.2017.01.006 28739214

[37] Alyamac Dizdar E Ozdemir R Sari FN Yurttutan S Gokmen T Erdeve O. Low platelet count is associated with ductus arteriosus patency in preterm newborns. Early Hum Dev. (2012) 88:813–6. 10.1016/j.earlhumdev.2012.05.007 22717423

[38] Sallmon H Metze B Koehne P Opgen-Rhein B Weiss K Will JC. Mature and immature platelets during the first week after birth and incidence of patent ductus arteriosus. Cardiol Young. (2020) 30:769–73. 10.1017/S1047951120000943 32340633

[39] Mirza H Garcia J McKinley G Hubbard L Sensing W Schneider J. Duration of significant patent ductus arteriosus and bronchopulmonary dysplasia in extremely preterm infants. J Perinatol. (2019) 39:1648–55. 10.1038/s41372-019-0496-5 31554913

[40] Philip R Waller B Chilakala S Graham B Stecchi N Apalodimas L. Hemodynamic and clinical consequences of early versus delayed closure of patent ductus arteriosus in extremely low birth weight infants. J Perinatol. (2020) 10.1038/s41372-020-00772-2 32792636

[41] deWaal K Phad N Collins N Boyle A. Cardiac remodeling in preterm infants with prolonged exposure to a patent ductus arteriosus. Congenit Heart Dis. (2017) 12:364–72. 10.1111/chd.12454 28225202

[42] Brown ER Increased risk of bronchopulmonary dysplasia in infants with patent ductus arteriosus. J Pediatr. (1979) 95:865–6. 10.1016/S0022-3476(79)80454-0 490263

[43] Lipman B Serwer GA Brazy JE. Abnormal cerebral hemodynamics in preterm infants with patent ductus arteriosus. Pediatrics. (1982) 69:778–81. 7079043

[44] Tomaszewska M Stork E Minich NM Friedman H Berlin S Hack M. Pulmonary hemorrhage: clinical course and outcomes among very low-birth-weight infants. Arch Pediatr Adolesc Med. (1999)153:715–721. 10.1001/archpedi.153.7.715 10401804

[45] Deshpande P Baczynski M McNamara PJ Jain A. Patent ductus arteriosus: the physiology of transition. Semin Fetal Neonatal Med. (2018) 23:225–31. 10.1016/j.siny.2018.05.001 29779927

[46] Benitz WE. Treatment of persistent patent ductus arteriosus in preterm infants: time to accept the null hypothesis? J Perinatol. (2010) 30:241–52. 10.1038/jp.2010.3 20182439

[47] Moodie DS Sathanandam S Qureshi AM Proceedings of the International PDA symposium. Congenit Heart Dis. (2019) 14:5–5. 10.1111/chd.12761 30811797

[48] Thébaud B Lacaze-Mazmonteil T. Patent ductus arteriosus in premature infants: a never-closing act. Paediatr Child Health. (2010) 15:267–70. 10.1093/pch/15.5.267 21532789 PMC2912622

[49] McNamara PJ Sehgal A. Towards rational management of the patent ductus arteriosus: the need for disease staging. Arch Dis Child Fetal Neonatal Ed. (2007) 92:F424–7. 10.1136/adc.2007.118117 17951547 PMC2675381

[50] Chock VY Punn R Oza A Benitz WE Van Meurs KP Whittemore AS Predictors of bronchopulmonary dysplasia or death in premature infants with a patent ductus arteriosus. Pediatr Res. (2014) 75:570–85. 10.1038/pr.2013.253 24378897 PMC3961500

[51] Mahony L Carnero V Brett C Heymann MA Clyman RI. Prophylactic indomethacin therapy for patent ductus arteriosus in very-low-birth-weight infants. N Engl J Med. (1982) 306:506–10. 10.1056/NEJM198203043060903 7035955

[52] Kluckow M Jeffery M Gill A Evans N. A randomized placebo-controlled trial of early treatment of the patent ductus arteriosus. Arch Dis Child Fetal Neonatal Ed. (2014) 99:F99–F104. 10.1136/archdischild-2013-304695 24317704

[53] Abdel-Hady H Nasef N Shabaan AE Nour I Patent ductus arteriosus in preterm infants: do we have the right answers? BioMed Res Int. (2013) 2013:676192 10.1155/2013/676192 24455715 PMC3885207

[54] Jain A Shah PS. Diagnosis, evaluation, and management of patent ductus arteriosus in preterm neonates. JAMA Pediatr. (2015) 169:863–872. 10.1001/jamapediatrics.2015.0987 26168357

[55] Van Laere D van Overmeire B Gupta S El-Khuffash A Savoia M McNamara PJ. Application of neonatologist performed echocardiography in the assessment of a patent ductus arteriosus. Pediatr Res. (2018) 84:46–56. 10.1038/s41390-018-0077-x 30072803 PMC6257219

[56] Paudel G Joshi V. Echocardiography of the patent ductus arteriosus in premature infant. Congenit Heart Dis. (2019) 14:42–5. 10.1111/chd.12703 30811799

[57] Shepherd JL Noori S. What is a hemodynamically significant PDA in preterm infants? Congenit Heart Dis. (2019) 14:21–6. 10.1111/chd.12727 30548469

[58] Sehgal A Menahem S Interparametric correlation between echocardio- graphic markers in preterm infants with patent ductus arteriosus. Pediatr Cardiol. (2013) 34:1212–7. 10.1007/s00246-013-0640-5 23370640

[59] Arlettaz R. Echocardiographic evaluation of patent ductus arteriosus in preterm infants. Front Pediatr. (2017) 5:147. 10.3389/fped.2017.00147.eCollection2017 28680875 PMC5478876

[60] Sehgal A McNamara PJ. Does echocardiography facilitate determination of hemodynamic significance attributable to the ductus arteriosus? Eur J Pediatr. (2009) 168:907–14. 10.1007/s00431-009-0983-3 19387684

[61] El Hajjar M Vaksmann G Rakza T Kongolo G Storme L. Severity of the ductal shunt: a comparison of different markers. Arch Dis Child Fetal Neonatal Ed. (2005) 90:F419–22. 10.1136/adc.2003.027698 16113155 PMC1721944

[62] Polat TB Celik IH Erdeve O. Early predictive echocardiographic features of hemodynamically significant patent ductus arteriosus in preterm VLBW infants. Pediatr Int. (2016) 58:589–94. 10.1111/ped.12915 26754187

[63] Kulkarni M Gokulakrishnan G Price J Fernandes CJ Leeflang M Pammi M. Diagnosing significant PDA using natriuretic peptides in preterm neonates: a systematic review. Pediatrics. (2015) 135:e510–25. 10.1542/peds.2014-1995 25601976

[64] El-Khuffash AF Molloy EJ. Influence of a patent ductus arteriosus on cardiac troponin T levels in preterm infants. J Pediatr. (2008) 153:350–3. 10.1016/j.jpeds.2008.04.014 18534211

[65] Slaughter JL Cua CL Notestine JL Rivera BK Marzec L Hade EM. Early prediction of spontaneous Patent Ductus Arteriosus (PDA) closure and PDA-associated outcomes: a prospective cohort investigation. BMC Pediatr. (2019) 19:333. 10.1186/s12887-019-1708-z 31519154 PMC6743099

[66] Celik IH Erdeve O Demirel G Canpolat FE Dilmen U. Elevated urinary NT-proBNP after pharmacological closure of patent ductus arteriosus in very low birth weight infants. Early Hum Dev. (2013) 89:187–9. 10.1016/j.earlhumdev.2012.09.020 23084575

[67] Alan S Karadeniz C Okulu E Kilic A Erdeve O Ucar T. Management of patent ductus arteriosus in preterm infants: clinical judgment might be a fair option. J Maternal-Fetal Neonatal Med. (2013) 26:1850–4. 10.3109/14767058.2013.801956 23650906

[68] Clyman RI Saha S Jobe A Oh W. Indomethacin prophylaxis for preterm infants: the impact of 2 multicentered randomized controlled trials on clinical practice. J Pediatr. (2007) 150:46–50. 10.1016/j.jpeds.2006.09.001 17188612 PMC1849955

[69] Benitz WE Bhombal S. The use of non-steroidal anti-inflammatory drugs for patent ductus arteriosus closure in preterm infants. Semin Fetal Neonatal Med. (2017) 22:302–7. 10.1016/j.siny.2017.07.004 28724506

[70] EL-Khuffash A Weisz DE McNamara PJ. Reflections of the changes in patent ductus arteriosus management during the last 10 years. Arch Dis Child Fetal Neonatal Ed. (2016) 101:F474–78. 10.1136/archdischild-2014-306214 27118761

[71] Fowlie PW Davis PG McGuire W Prophylactic intravenous indomethacin for preventing mortality and morbidity in preterm infants. Cochrane Database Syst Rev. (2010) CD000174. 10.1002/14651858.CD000174.pub2 PMC7045285 20614421

[72] Schmidt B Asztalos EV Roberts RS Robertson CM Sauve RS Whitfield MF. Impact of bronchopulmonary dysplasia, brain injury, and severe retinopathy on the outcome of extremely low-birth-weight infants at 18 months: results from the trial of indomethacin prophylaxis in preterms. JAMA. (2003) 289:1124–9. 10.1001/jama.289.9.1124 12622582

[73] Ment LR Vohr B Allan W Westerveld M Sparrow SS Schneider KC. Outcome of children in the indomethacin intraventricular hemorrhage prevention trial. Pediatrics. (2000) 105:485–91. 10.1542/peds.105.3.485 10699097

[74] Clyman RI Liebowitz M Kaempf J Erdeve O Bulbul A Håkansson S. PDA-TOLERATE trial: an exploratory randomized controlled trial of treatment of moderate-to-large patent ductus arteriosus at 1 week of age. J. Pediatr. (2019) 205:41–8.e6. 10.1016/j.jpeds.2018.09.012 30340932 PMC6502709

[75] Okulu E Erdeve O Arslan Z Demirel N Kaya H Kursad I. An Observational, prospective, multicenter, registry-based cohort study comparing conservative and medical management for patent ductus arteriosus. Front Pediatr. (2020) 8:434. 10.3389/fped.2020.00434 32850547 PMC7411351

[76] Liebowitz M Kaempf J Erdeve O Bulbul A Håkansson S Lindqvist J. Comparative effectiveness of drugs used to constrict the patent ductus arteriosus: a secondary analysis of the PDA-TOLERATE trial (NCT01958320). J Perinatol. (2019) 39:599–607. 10.1038/s41372-019-0347-4 30850756 PMC6561645

[77] Cassady G Crouse DT Kirklin JW Strange MJ Joiner CH Godoy G A randomized, controlled trial of very early prophylactic ligation of the ductus arteriosus in babies who weighed 1000 g or less at birth. N Engl J Med. (1989) 320:1511–6. 10.1056/NEJM198906083202302 2498657

[78] Mikhail M Lee W Toews W Synhorst DP Hawes CR Hernandez J. Surgical and medical experience with 734 premature infants with patient ductus arteriosus. J Thorac Cardiovasc Surg. (1982) 83:349–57. 10.1016/S0022-5223(19)37268-X 7062747

[79] El-Khuffash AF Jain A McNamara PJ. Ligation of the patent ductus arteriosus in preterm infants: understanding the physiology. J Pediatr. (2013) 162:1100–6. 10.1016/j.jpeds.2012.12.094 23410600

[80] US Food and Drug Administration (FDA) Sterile Indocin1 I.V. (Indomethacin for Injection). FDA Label. (2009). (2014). Available online at: http://www.accessdata.fda.gov/drugsatfda_docs/label/2010/018878s027lbl.pdf (accessed November 6, 2014).

[81] US Food and Drug Administration (FDA) NeoProfen1 (ibuprofen lysine) Injection. NDA 21-903; Approval Letter. April (2006). Available online at: http://www.accessdata.fda.gov/drugsatfda_docs/label/2006/021903lbl.pdf (accessed November 7, 2014).

[82] Ohlsson A Walia R Shah SS Ibuprofen for the treatment of patent ductus arteriosus in preterm or low birth weight (or both) infants. Cochrane Database Syst Rev. (2015) CD003481. 10.1002/14651858.CD003481.pub6 25692606

[83] van Overmeire B Touw D Schepens PJ Kearns GL van den Anker JN. Ibuprofen pharmacokinetics in preterm infants with patent ductus arteriosus. Clin Pharmacol Ther. (2001) 70:336–43. 10.1067/mcp.2001.118453 11673749

[84] Hirt D van Overmeire B Treluyer JM Langhendries JP Marguglio A Eisinger MJ. An optimized ibuprofen dosing scheme for preterm neonates with patent ductus arteriosus, based on a population pharmacokinetic and pharmacodynamic study. Br J Clin Pharmacol. (2008) 65:629–36. 10.1111/j.1365-2125.2008.03118.x 18307541 PMC2432471

[85] Desfrere L Zohar S Morville P Brunhes A Chevret S Pons G Moriette G Rey E Treluyer JM. Dose-finding study of ibuprofen in patent ductus arteriosus using the continual reassessment method. J Clin Pharm Ther. (2005) 30:121–32. 10.1111/j.1365-2710.2005.00630.x 15811164

[86] Mitra S Florez ID Tamayo ME. Association of placebo, indomethacin, ibuprofen, and acetaminophen with closure of hemodynamically significant patent ductus arteriosus in preterm infantsA systematic review and meta-analysis. JAMA. (2018) 319:1221–38. 10.1001/jama.2018.1896 29584842 PMC5885871

[87] Kanmaz G Erdeve O Canpolat FE Oguz SS Uras N Altug N. Serum ibuprofen levels of extremely preterm infants treated prophylactically with oral ibuprofen to prevent patent ductus arteriosus. Eur J Clin Pharmacol. (2013) 69:1075201381. 10.1007/s00228-012-1438-8 23128963

[88] Yurttutan S Oncel MY Dilmen U Erdeve O Ozdemir R. The relationship between trough drug concentrations and ductal closure in preterm infants treated with three-dose-oral ibuprofen. J Matern Fetal Neonatal Med. (2013) 26:1306–10. 10.3109/14767058.2013.784739 23488980

[89] Pacifici GM Clinical Pharmacology of Ibuprofen in Preterm Infants: A Meta-Analysis of Published Data. Available online at: http://www.gnresearch.org/doi/10.5935/MedicalExpress.2014.02.02 (accessed September 30, 2016).

[90] Erdeve O Yurttutan S Altug N Ozdemir R Gokmen T Dilmen U. Oral versus intravenous ibuprofen for patent ductus arteriosus closure: a randomised controlled trial in extremely low birthweight infants. Arch Dis Child Fetal Neonatal Ed. (2012) 97:F279–83. 10.1136/archdischild-2011-300532 22147286

[91] Gokmen T Erdeve O Altug N Oguz SS Uras N Dilmen U. Efficacy and safety of oral versus intravenous ibuprofen in very low birth weight preterm infants with patent ductus arteriosus. J Pediatr. (2011) 158:549–54.e1. 10.1016/j.jpeds.2010.10.008 21094951

[92] Eras Z Gokmen T Erdeve O Ozyurt BM Saridas B Dilmen U. Impact of oral versus intravenous ibuprofen on neurodevelopmental outcome: a randomized controlled parallel study. Am J Perinatol. (2013) 30:857–62. 10.1055/s-0033-1333667 23359230

[93] Pezzati M Vangi V Biagiotti R Bertini G Cianciulli D Rubaltelli FF. Effects of indomethacin and ibuprofen on mesenteric and renal blood flow in preterm infants with patent ductus arteriosus. J Pediatr. (1999) 135:733–8. 10.1016/S0022-3476(99)70093-4 10586177

[94] Patel J Roberts I Azzopardi D Hamilton P Edwards AD. Randomized double-blind controlled trial comparing the effects of ibuprofen with indomethacin on cerebral hemodynamics in preterm infants with patent ductus arteriosus. Pediatr Res. (2000) 47:36–42. 10.1203/00006450-200001000-00009 10625080

[95] Guzoglu N Sari FN Ozdemir R Oguz SS Uras N Altug N. Renal and mesenteric tissue oxygenation in preterm infants treated with oral ibuprofen. J Matern Fetal Neonatal Med. (2014) 27:197–203. 10.3109/14767058.2013.811485 23735121

[96] Van Bel F Van Zwieten PH Den Ouden LL. Contribution of color Doppler flow imaging to the evaluation of the effect of indomethacin on neonatal cerebral hemodynamics. J Ultrasound Med. (1990) 9:107–9. 10.7863/jum.1990.9.2.107 2137182

[97] Van Bel F Van Zoeren D Schipper J Guit GL Baan J. Effect of indomethacin on superior mesenteric artery blood flow velocity in preterm infants. J Pediatr. (1990) 116:965–70. 10.1016/S0022-3476(05)80662-6 2112189

[98] Clyman R Wickremasinghe A Jhaveri N Hassinger DC Attridge JT Sanocka U. Enteral feeding during indomethacin and ibuprofen treatment of a patent ductus arteriosus. J Pediatr. (2013) 163:406–11. 10.1016/j.jpeds.2013.01.057 23472765 PMC3683087

[99] Yanowitz TD Reese J Gillam-Krakauer M Cochran CM Jegatheesan P Lau J. Superior mesenteric artery blood flow velocities following medical treatment of a patent ductus arteriosus. J Pediatr. (2014) 164:661–3. 10.1016/j.jpeds.2013.11.002 24321538 PMC4077598

[100] Ferguson JM. Pharmacotherapy for patent ductus arteriosus closure. Congenit Heart Dis. (2019) 14:52–6. 10.1111/chd.12715 30536827

[101] Oncel MY Yurttutan S Erdeve O Uras N Altug N Oguz SS. Oral paracetamol versus oral ibuprofen in the management of patent ductus arteriosus in preterm infants: a randomized controlled trial. J Pediatr. (2014) 164:510–4.e1. 10.1016/j.jpeds.2013.11.008 24359938

[102] Ohlsson A Shah PS Paracetamol (acetaminophen) for patent ductus arteriosus in preterm or low-birth-weight infants. Cochrane Database Syst Rev. (2015) CD010061. 10.1002/14651858.CD010061.pub2 25758061

[103] Yurttutan S Oncel MY Arayici S Uras N Altug N Erdeve O. A different first-choice drug in the medical management of patent ductus arteriosus: oral paracetamol. J Matern Fetal Neonatal Med. (2013) 26:825–7. 10.3109/14767058.2012.755162 23205872

[104] Oncel MY Erdeve O. Oral medications regarding their safety and efficacy in the management of patent ductus arteriosus. World J Clin Pediatr. (2016). 5:75–81. 10.5409/wjcp.v5.i1.75 26862505 PMC4737696

[105] Oncel MY Erdeve O. Safety of therapeutics used in management of patent ductus arteriosus in preterm infants. Curr Drug Saf . (2015) 10:106–112. 10.2174/1574886309999141030142847 25323589

[106] Terrin G Conte F Oncel MY Scipione A McNamara PJ Simons S. Paracetamol for the treatment of patent ductus arteriosus in preterm neonates: a systematic review and meta-analysis. Arch Dis Child Fetal Neonatal Ed. (2016). 101:F127–36. 10.1136/archdischild-2014-307312 26283668

[107] Davidson JM Ferguson J Ivey E Philip R Weems MF Talati AJ. A randomized trial of intravenous acetaminophen versus indomethacin for treatment of hemodynamically significant PDAs in VLBW infants. J Perinatol. (2020). 10.1038/s41372-020-0694-1 32439957

[108] Oncel MY Eras Z Uras N Canpolat FE Erdeve O Oguz SS. Neurodevelopmental outcomes of preterm infants treated with oral paracetamol versus ibuprofen for patent ductus arteriosus. Am J Perinatol. (2017) 34:1185–9. 10.1055/s-0037-1601564 28395364

[109] Yurttutan S Bozkaya A Hüdayioglu F Oncel MY. The effect of combined therapy for treatment of monotherapy-resistant PDA in preterm infants. J Matern Fetal Neonatal Med. (2019) 32:3662–5. 10.1080/14767058.2018.1481043 29921134

[110] Hochwald O Mainzer G Borenstein-Levin L Jubran H Dinur G Zucker M. Adding paracetamol to Ibuprofen for the treatment of patent ductus arteriosus in preterm infants: a double-blind, randomized, placebo-controlled pilot study. Am J Perinatol. (2018) 35:1319–25. 10.1055/s-0038-1653946 29783269

[111] Cotton RB Stahlman MT Bender HW Graham TP Catterton WZ Kovar I. Randomized trial of early closure of symptomatic patent ductus arteriosus in small preterm infants. J Pediatr. (1978) 93:647–51. 702245 10.1016/s0022-3476(78)80910-x

[112] Raval MV Laughon MM Bose CL Phillips JD. Patent ductus arteriosus ligation in premature infants: who really benefits, and at what cost? J Pediatr Surg. (2007) 42:69–75. 10.1016/j.jpedsurg.2006.09.040 17208543

[113] Madan JC Kendrick D Hagadorn JI Frantz ID 3rd. Patent ductus arteriosus therapy: impact on neonatal and 18-month outcome. Pediatrics. (2009) 123:674–81. 10.1542/peds.2007-2781 19171637 PMC2752886

[114] Mirea L Sankaran K Seshia M Ohlsson A Allen AC Aziz K. Treatment of patent ductus arteriosus and neonatal mortality/morbidities: adjustment for treatment selection bias. J Pediatr. (2012) 161:689–94. 10.1016/j.jpeds.2012.05.007 22703954

[115] Janz-Robinson EM Badawi N Walker K Bajuk B Abdel-Latif ME. Neonatal intensive care units network. Neurodevelopmental outcomes of premature infants treated for patent ductus arteriosus: a population-based cohort study. J Pediatr. (2015) 167:1025–32. 26227439 10.1016/j.jpeds.2015.06.054

[116] Weisz DE More K McNamara PJ Shah PS. PDA ligation and health outcomes: a meta-analysis. Pediatrics. (2014) 133:e1024–46. 10.1542/peds.2013-3431 24639268

[117] Benjamin JR Smith PB Cotten CM Jaggers J Goldstein RF Malcolm WF. Long-term morbidities associated with vocal cord paralysis after surgical closure of a patent ductus arteriosus in extremely low birth weight infants. J Perinatol. (2010) 30:408–13. 10.1038/jp.2009.124 19759545 PMC2878380

[118] Chen H Weng G Chen Z Wang H Xie Q Bao J. Comparison of posterolateral thoracotomy and video-assisted thoracoscopic clipping for the treatment of patent ductus arteriosus in neonates and infants. Pediatr Cardiol. (2011) 32:386–90. 10.1007/s00246-010-9863-x 21188372

[119] Clement WA El-Hakim H Phillipos EZ Cote JJ. Unilateral vocal cord paralysis following patent ductus arteriosus ligation in extremely low-birth-weight in- fants. Arch Otolaryngol Head Neck Surg. (2008) 134:28–33. 10.1001/archoto.2007.2 18209132

[120] Hsu KH Chiang MC Lien R Chu JJ Chang YS Chu SM. Diaphragmatic paralysis among very low birth weight infants following ligation for patent ductus arteriosus. Eur J Pediatr. (2012) 171:1639–44. 10.1007/s00431-012-1787-4 22763604

[121] Mandhan P Brown S Kukkady A Samarakkody U. Surgical closure of patent ductus arteriosus in preterm low birth weight infants. Congenit Heart Dis. (2009) 4:34–7. 10.1111/j.1747-0803.2008.00241.x 19207401

[122] Smith ME King JD Elsherif A Muntz HR Park AH Kouretas PC. Should all newborns who undergo patent ductus arteriosus ligation be examined for vocal fold mobility? Laryngoscope. (2009) 119:1606–9. 10.1002/lary.20148 19507238

[123] Spanos WC Brookes JT Smith MC Burkhart HM Bell EF Smith RJ. Unilateral vocal fold paralysis in premature infants after ligation of patent ductus arteriosus: vascular clip versus suture ligature. Ann Otol Rhinol Laryngol. (2009) 118:750–3. 10.1177/000348940911801011 19894404

[124] Baruteau AE Hascoët S Baruteau J Boudjemline Y Lambert V Angel CY. Transcatheter closure of patent ductus arteriosus: past, present and future. Arch Cardiovasc Dis. (2014) 107:122–32. 10.1016/j.acvd.2014.01.008 24560920

[125] Backes CH Rivera BK Bridge JA Armstrong AK Boe BA Berman DP. Percutaneous patent ductus arteriosus (PDA) closure during infancy: a meta-analysis. Pediatrics. (2017) 139:e20162927. 10.1542/peds.2016-2927 28087683

[126] Zahn EM Peck D Phillips A Nevin P Basaker K Simmons C. Transcatheter closure of patent ductus arteriosus in extremely premature newborns: early results and midterm follow-up. JACC Cardiovasc Interv. (2016) 9:2429–37. 10.1016/j.jcin.2016.09.019 27931595

[127] Sathanandam S Balduf K Chilakala S Washington K Allen K Knott-Craig C. Role of transcatheter patent ductus arteriosus closure in extremely low birth weight infants. Catheter Cardiovasc Interv. (2019) 93:89–6. 10.1002/ccd.27808 30269408

[128] Agrawal H Parkerson S Lighter D Fowke J Goedecke P Towbin J Transcatheter closure of PDA compared to surgical ligation in premature neonates: clinical outcomes and cost comparison. Catheter Cardiovasc Interv. (2020) 95:1–229. 10.12669/pjms.324.10048 31609084

[129] Willis A Pereiras L Head T Dupuis G Sessums J Corder G. Transport of extremely low birth weight neonates for persistent ductus arteriosus closure in the catheterization lab. Congenit Heart Dis. (2019) 14:69–73. 10.1111/chd.12706 30811788

[130] Tadphale S Yohannan T Kauffmann T Maller V Agrawal V Lloyd H. Accessing femoral arteries less than 3 mm in diameter is associated with increased incidence of loss of pulse following cardiac catheterization in infants. Pediatr Cardiol. (2020) 41:1058–66. 10.1007/s00246-020-02357-4 32367307

[131] Alexander J Yohannan T Abutineh I Agrawal V Lloyd H Zurakowski D. Ultrasound-guided femoral arterial access in pediatric cardiac catheterizations: a prospective evaluation of the prevalence, risk factors, and mechanism for acute loss of arterial pulse. Catheter Cardiovasc Interv. (2016) 88:1098–107. 10.1002/ccd.26702 27535615

[132] Tadphale SD Zurakowski D Bird LE Yohannan TM Agrawal VK Lloyd H. Construction of femoral vessel nomograms for planning cardiac interventional procedures in children 0-4 years old. Pediatr Cardiol. (2020) 41:1135–44. 10.1007/s00246-020-02363-6 32363434

[133] Sathanandam S Agrawal H Chilakala S Johnson J Allen K Knott-Craig C. Can transcatheter PDA closure be performed in neonates ≤ 1,000 grams? The Memphis experience. Congenit Heart Dis. (2019) 14:79–84. 10.1111/chd.12700 30811793

[134] Sathanandam S Justino H Waller BR 3rd Radke W Qureshi A Initial clinical experience with the medtronic micro vascular plug^TM^ in transcatheter occlusion of PDAs in extremely premature infants. Catheter Cardiovasc Interv. (2017) 89:1051–8. 10.1002/ccd.26878 27888552

[135] Johnson JN Sathanandam S Naik R Philip R Echocardiographic guidance for transcatheter patent ductus arteriosus closure in extremely low birth weight infants. Congenit Heart Dis. (2019) 14:74–8. 10.1111/chd.12725 30811801

[136] Sathanandam S Gianinni A Sefton E Greer K Stecchi N Allen K. Live broadcast of transcatheter PDA closure in a 700 grams ELBW infant during the international PDA symposium. Congenit Heart Dis. (2019) 14:85–9. 10.1111/chd.12710 30811797

[137] Agrawal H Waller BR Surendan S Sathanandam S. New patent ductus arteriosus closure devices and techniques. Interv Cardiol Clin. (2019) 8:23–32. 10.1016/j.iccl.2018.08.004 30449419

[138] Sathanandam SK Gutfinger D O'Brien L Forbes TJ Gillespie MJ Berman DP. Amplatzer Piccolo Occluder clinical trial for percutaneous closure of the patent ductus arteriosus in patients ≥700 grams. Catheter Cardiovasc Interv. (2020) 96:1266–76. 10.1002/ccd.28973 32433821 PMC7754477

[139] Apalodimas L Waller BR Philip R Crawford J Cunningham J Sathanandam S. A comprehensive program for preterm infants with patent ductus arteriosus. Congenit Heart Dis. (2019) 14:90–4. 10.1111/chd.12705 30811791

[140] Sathanandam S Apalodimas L Weems M Rush Waller B Philip R Establishing a Robust Transcatheter PDA Closure Program for Extremely Low Birth Weight Infants. Congenital Cardiology Today – March (2018). Issue featured article. ISSN 1554-7787; ISSN 1554-0499.

[141] Kimball TR Ralston MA Khoury P Crump RG Cho FS Reuter JH. Effect of ligation of patent ductus arteriosus on left ventricular performance and its determinants in premature neonates. J Am Coll Cardiol. (1996) 27:193–7. 10.1016/0735-1097(95)00452-1 8522694

[142] Ulrich TJB Hansen TP Reid KJ Bingler MA Olsen SL. Post-ligation cardiac syndrome is associated with increased morbidity in preterm infants. J Perinatol. (2018) 38:537–42. 10.1038/s41372-018-0056-4 29453434

[143] McNamara PJ Stewart L Shivananda SP Stephens D Sehgal A. Patent ductus arteriosus ligation is associated with impaired left ventricular systolic performance in premature infants weighing less than 1000 g. J Thorac Cardiovasc Surg. (2010) 140:150–7. 10.1016/j.jtcvs.2010.01.011 20363478

[144] Philip R Waller B Chilakala S Apalodimas L Sathanandam S Comparison of low cardiac output syndrome after PDA ligation and transcatheter PDA closure in extremely low birth weight infants. J Am Coll Cardiol. (2019) 73(9 Suppl.):575. 10.1016/S0735-1097(19)31183-0

[145] Serrano RM Madison M Lorant D Hoyer M Alexy R. Comparison of 'post-patent ductus arteriosus ligation syndrome' in premature infants after surgical ligation vs. percutaneous closure. J Perinatol. (2020) 40:324–9. 10.1038/s41372-019-0513-8 31578421

[146] Harvey E Philip R Waller B Johnson J Cunningham J Chilakala S Scoring system for post ligation cardiac syndrome and its utility after transcatheter and surgical patent ductus arteriosus ligation in extremely low birthweight infants. Circulation. (2019) 140:A14799 Available online at: https://www.ahajournals.org/doi/abs/10.1161/circ.140.suppl_1.14799?af=R

[147] Jain A Sahni M El-Khuffash A Khadawardi E Sehgal A McNamara PJ. Use of targeted neonatal echocardiography to prevent postoperative cardiorespiratory instability after patent ductus arteriosus ligation. J Pediatr. (2012) 160:584–9. 10.1016/j.jpeds.2011.09.027 22050874

[148] Tailor N Philip R Harvey E Dupuis G Waller B Stecchi N Effect of intravenous calcium administration during transcatheter PDA closure in ELBW infants. Catheter Cardiovasc Interv. (2020) 95:1–229. 10.1002/ccd.28864 31609084

